# Understanding Users’ Vaping Experiences from Social Media: Initial Study Using Sentiment Opinion Summarization Techniques

**DOI:** 10.2196/jmir.9373

**Published:** 2018-08-15

**Authors:** Qiudan Li, Can Wang, Ruoran Liu, Lei Wang, Daniel Dajun Zeng, Scott James Leischow

**Affiliations:** ^1^ The State Key Laboratory of Management and Control for Complex Systems Institute of Automation, Chinese Academy of Sciences Beijing China; ^2^ University of Chinese Academy of Sciences Beijing China; ^3^ Department of Management Information Systems Eller College of Management The University of Arizona Tucson, AZ United States; ^4^ College of Health Solutions Arizona State University Phoenix, AZ United States

**Keywords:** electronic nicotine delivery systems, e-cigarette, e-liquid, JuiceDB, sentiment opinion summarization, social media, vaping, infodemiology

## Abstract

**Background:**

E-liquid is one of the main components in electronic nicotine delivery systems (ENDS). ENDS review comments could serve as an early warning on use patterns and even function to serve as an indicator of problems or adverse events pertaining to the use of specific e-liquids—much like types of responses tracked by the Food and Drug Administration (FDA) regarding medications.

**Objective:**

This study aimed to understand users’ “vaping” experience using sentiment opinion summarization techniques, which can help characterize how consumers think about specific e-liquids and their characteristics (eg, flavor, throat hit, and vapor production).

**Methods:**

We collected e-liquid reviews on JuiceDB from June 27, 2013 to December 31, 2017 using its public application programming interface. The dataset contains 27,070 reviews for 8058 e-liquid products. Each review is accompanied by an overall rating and a set of 4 aspect ratings of an e-liquid, each on a scale of 1-5: flavor accuracy, throat hit, value, and cloud production. An iterative dichotomiser 3 (ID3)-based influential aspect analysis model was adopted to learn the key elements that impact e-liquid use. Then, fine-grained sentiment analysis was employed to mine opinions on various aspects of vaping experience related to e-liquids.

**Results:**

We found that flavor accuracy and value were the two most important aspects that affected users’ sentiments toward e-liquids. Of reviews in JuiceDB, 67.83% (18,362/27,070) were positive, while 12.67% (3430/27,070) were negative. This indicates that users generally hold positive attitudes toward e-liquids. Among the 9 flavors, fruity and sweet were the two most popular. Great and sweet tastes, reasonable value, and strong throat hit made users satisfied with fruity and sweet flavors, whereas “strange” tastes made users dislike those flavors. Meanwhile, users complained about some e-liquids’ steep or expensive prices, bad quality, and harsh throat hit. There were 2342 fruity e-liquids and 2049 sweet e-liquids. There were 55.81% (1307/2342) and 59.83% (1226/2049) positive sentiments and 13.62% (319/2342) and 12.88% (264/2049) negative sentiments toward fruity e-liquids and sweet e-liquids, respectively. Great flavors and good vapors contributed to positive reviews of fruity and sweet products. However, bad tastes such as “sour” or “bitter” resulted in negative reviews. These findings can help businesses and policy makers to further improve product quality and formulate effective policy.

**Conclusions:**

This study provides an effective mechanism for analyzing users’ ENDS vaping experience based on sentiment opinion summarization techniques. Sentiment opinions on aspect and products can be found using our method, which is of great importance to monitor e-liquid products and improve work efficiency.

## Introduction

The market for electronic nicotine delivery systems (ENDS) or electronic cigarettes (e-cigarettes) is growing rapidly. According to data from Research and Markets, the global electronic cigarette market was expected to grow at a compound annual rate of 16.6% during 2017-2022 and hit US $27.7 billion by 2022 [1]. E-cigarettes are now the most commonly used tobacco product among youth [2]. More than 2 million middle and high school students used e-cigarettes in 2016 [3]. Among middle school students, 31% use e-cigarettes because they contain multiple flavors [4]. To protect Americans from dangers of tobacco and nicotine, the US Food and Drug Administration (FDA) extended its authority to e-cigarettes in 2016 [5]. ENDS or e-cigarette products heat a liquid (e-liquid) that may contain nicotine, as well as varying compositions of flavorings, propylene glycol (PG), vegetable glycerin (VG), and other ingredients into an aerosol that the user inhales [3]. All elements in the e-liquid form the unique “vaping” experience. For example, VG produces more vapor than PG and offers a slight sweetness; PG provides more “throat hit” and usually carries a stronger flavor [6-9]. Mining vaping experience with e-liquid products can help FDA policy makers understand use patterns and reasons users like or dislike products and thus make better decisions.

Social media such as Facebook, Twitter, and YouTube have recently become significant platforms for health surveillance and social intelligence [10,11], also providing new insights on e-cigarettes to help inform future research, regulations, surveillance, and enforcement efforts [12]. For example, Liang et al studied the prevalence and promotional strategies of protobacco content in Facebook, Wikipedia, and YouTube [13]. Chu et al examined marketing strategies of leading e-cigarette brands on multiple social networking sites including Facebook, Twitter, Google+, and Instagram [14]. Hua et al showed that e-cigarette use can have wide-ranging positive and negative effects by analyzing health effects in Web-based forums [15]. Kim et al used Twitter data to gain insights into e-cigarette marketing and locations of use [12]. Cole-Lewis et al conducted content analysis to identify key conversation trends and patterns over time using historical Twitter data [16]. Cole-Lewis et al adopted supervised machine learning-based predictive classification models to assess Twitter data for a range of e-cigarette-related factors, thus helping the development of public health communication, policies, and interventions regarding e-cigarettes [17]. Lazard et al uncovered key patterns and important e-cigarette topics on Twitter [18]. Harris et al analyzed messages and tweet patterns to mine the response to the Chicago Department of Public Health’s e-cigarette campaign [19]. Huang et al quantified e-cigarette-related videos on YouTube, assessed their content, and characterized levels of engagement with the videos [20].

As two new social media platforms, Reddit and JuiceDB have been studied to analyze broadly discussed vaping methods and features including flavor, throat hit, and vapor production. In previous research, Wang et al have identified 8 categories of flavors on Reddit: fruits, cream, tobacco, menthol, beverages, sweet, seasonings, and nuts [21]. In JuiceDB, there were 9 flavor categories: sweet, fruity, rich, creamy, spiced, tobacco, cool, nutty, and coffee. The two category systems were fairly consistent, providing a good schema for future research. Li et al mined potential relationships between symptoms and e-liquid components, such as PG, VG, flavor extracts, and nicotine, using user-generated data collected from Reddit [22]. Jin et al performed e-liquid review rating prediction by jointly modeling review content and aspect ratings [23]. Zhan et al examined Reddit, JuiceDB, and Twitter as social media data sources for e-cigarette research and adopted latent Dirichlet allocation topic modeling techniques to identify 4 topics across platforms: promotions, flavor discussions, experience sharing, and regulation debates [24]. Chen et al analyzed polarities of e-liquid features by mining Web-based reviews [25]. These studies showed the importance of flavor in ENDS or e-cigarette products.

Despite the growing amount of literature regarding ENDS or e-cigarettes on social media, to date, no published studies have systematically mined users’ e-liquid usage patterns from review data based on opinion summarization techniques. JuiceDB [26], one of the world’s largest independent e-liquid and vape juice resources, has great influence in promoting e-liquid product use through user-generated content, that is, it allows users to share their vaping experience with different e-liquid products by leaving detailed comments, aspect ratings, and overall product ratings. This study aims to answer the following questions. Which factors have the most influence on users’ sentiments toward e-liquids? What are the most popular flavors? Why do users like flavors and products? Data-driven findings could serve as an early warning on use patterns and even function to indicate problems or adverse events pertaining to use of specific e-liquids.

## Methods

### Framework

**Figure 1:**
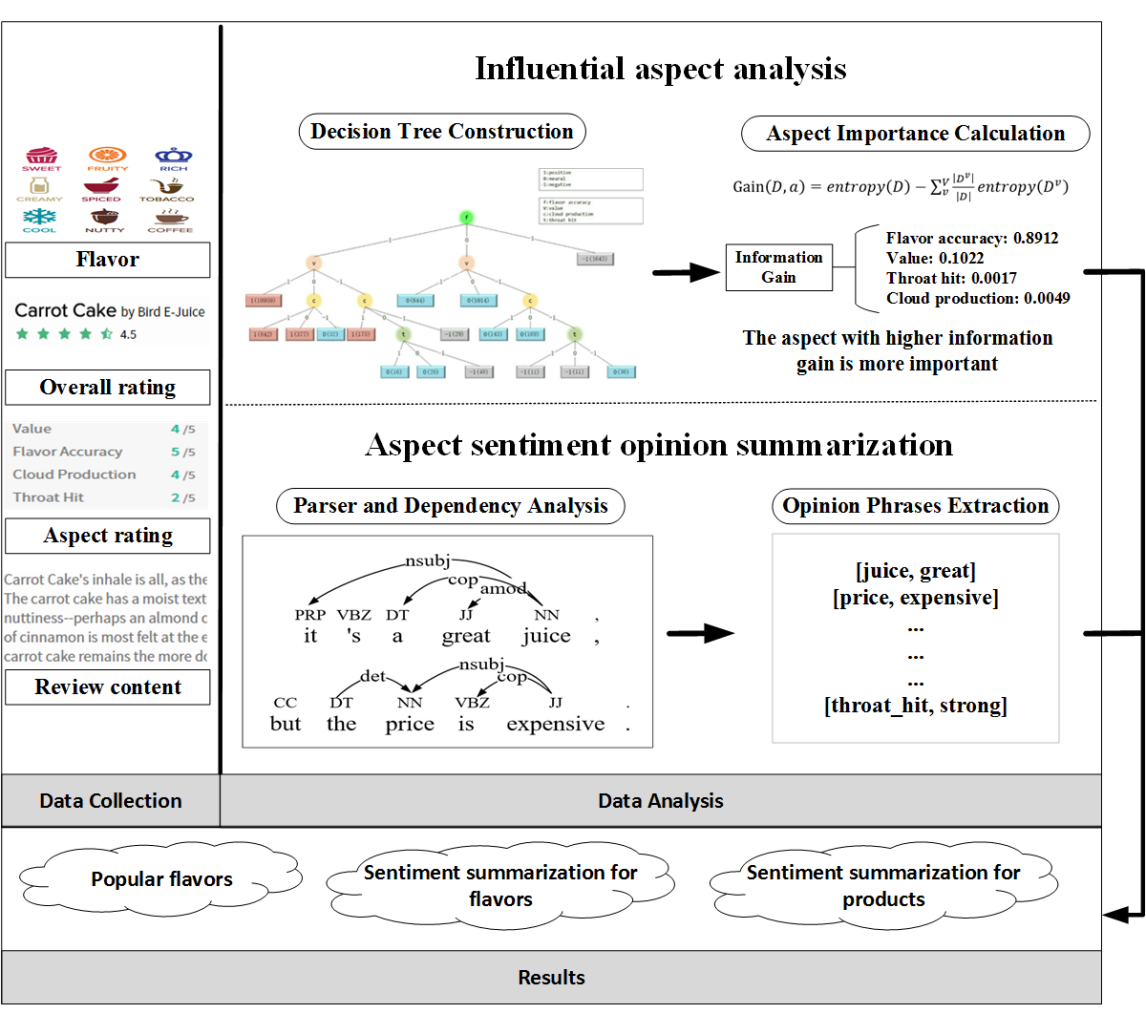
The framework to analyze users' electronic nicotine delivery systems vaping experience. Amod: adjectival modifier dependency relationship; CC: coordinating conjunction; cop: copula dependency relationship; det: determiner dependency relationship; DT: determiner; JJ: adjective; NN: noun, singular or mass; nsubj: nominal subject dependency relationship; PRP: personal pronoun; VBZ: verb, third person singular present.

Figure 1 shows the framework for analyzing users’ ENDS vaping experience. It consists of three components: data collection and preprocessing, data analysis, and results.

### Data Collection and Preprocessing

Since the first review by JuiceDB’s API was published on June 27, 2013, we used API to collect e-liquid reviews on JuiceDB, one of the world’s largest independent e-liquid and vape juice resources, from June 27, 2013 to December 31, 2017. The JuiceDB website provides flavor category information for each product. Registered users can provide reviews for e-liquids consisting of an overall rating and aspect ratings that respectively reflect their sentiments toward the product and its attributes. Each review is accompanied by an overall rating and a set of 4 e-liquid aspect ratings on a scale from 1 to 5: flavor accuracy, throat hit, value, and cloud production. The dataset contains 27,070 reviews for 8058 e-liquid products.

To better understand the sentiment toward products and aspects, discretization processing is necessary. A positive label is given to a product or aspect if the overall rating or aspect rating is ≥4; a neutral label is generated if the rating score is ≥3 and <4; and a negative label is assigned if the score is <3.

### Data Analysis

This study aimed to understand users’ e-liquid usage patterns by mining summarization, which helps explain reasons users like or dislike a product. The following processes were performed.

#### Influential Aspect Analysis

To evaluate the importance of aspects that influence a user’s sentiment toward an e-liquid product, the iterative dichotomiser 3 (ID3) algorithm was adopted to construct a decision tree, which has turned out to be an efficient method of identifying important features [27]. The key idea of the method was to compute feature importance based on information gain. An aspect with higher information gain has greater influence on users’ sentiments toward an e-liquid product. First, both aspect ratings and overall ratings were discretized. Second, the ID3 algorithm computed the information gain of each aspect and split the dataset into subsets according to the value of the aspect with the largest information gain. This process was iterated on each subset until there was no available aspect. Finally, the importance of an aspect was computed as the normalized total information gain brought by the corresponding aspect. The aspect with a higher value was considered more important.

#### Aspect Sentiment Opinion Summarization

Opinions are aspect-sentiment pairs that summarize a user’s sentiment toward a product at a fine granularity. Opinion summarization modeling aims to automatically mine aspect words and their corresponding sentiment words [28]. The model consists of the following two steps.

##### Step 1: Parser and Dependency Analysis

To identify words’ part-of-speech tag and dependency in review sentences, Stanford Parser 3.4 [29], one module in the Stanford natural language processing toolbox, was adopted. For example, in “Flavor is great, definitely an adv,” the adjective “great” modifies the noun “flavor.”

##### Step 2: Opinion Phrases Extraction

Based on the above results, aspect-sentiment pairs were extracted. An aspect word is usually a noun. Term frequency was adopted to measure the importance of nouns, and we selected nouns whose term frequency was >20 as candidate words. Then, meaningful aspect words were manually selected. Sentiments are adjective words that modify the aspect words. The sentiment polarity of aspect-sentiment pairs was identified by the popular emotional word dictionary [30]. For example, an opinion phrase “great flavor” can be extracted from “A great flavor. Tastes like tobacco with waffles and maple syrup,” and the corresponding sentiment polarity is positive.

## Results

### Influential Aspect Analysis

Aspect ratings such as flavor accuracy, throat hit, value, and cloud production reflect users’ feelings about more specific aspects of an e-liquid product. The overall rating score is a mixture of product quality and the customer’s overall interest in the product. Analyzing the relationship between aspect ratings and overall rating can help identify important aspects that influence users’ interest in a product and impact marketing or product decisions.

**Figure 2:**
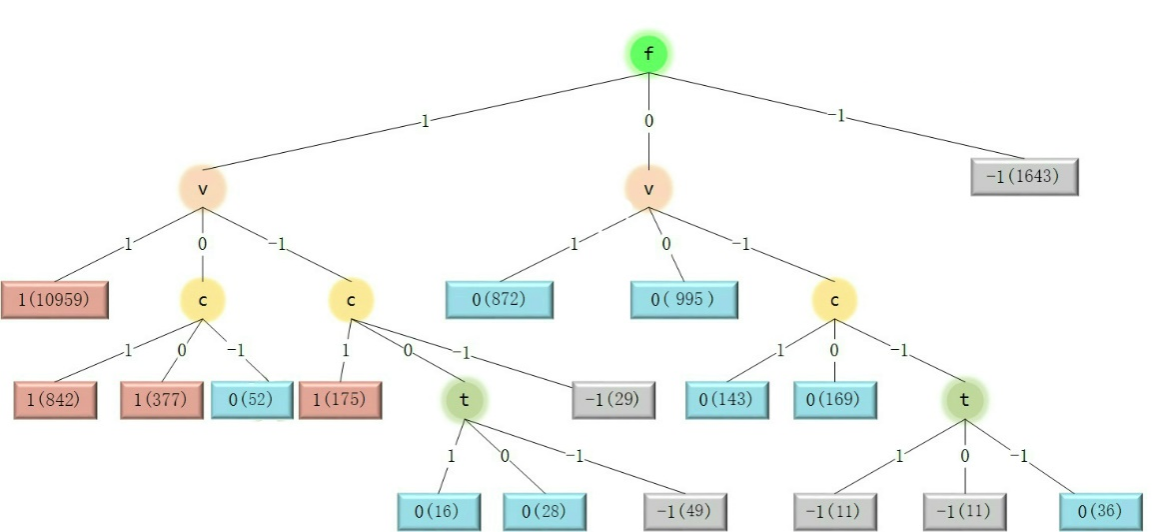
The decision tree constructed on reviews without missing aspect ratings. C: cloud production; f: flavor accuracy; t: throat hit; v: value; 1: positive; 0: neutral; -1: negative.

Influential aspect analysis was performed on 16,407 reviews with no missing aspect ratings. The decision tree constructed in this analysis process is shown in Figure 2. Specifically, the label on the branch node means that this dataset is split into subsets according to the corresponding aspect. For example, the label “f” on the root node meant that the dataset was split into 3 subsets according to the value of the flavor accuracy aspect. The label on the edge from a parent node to a child node represented a condition. As another example, the label “1” on the edge from the root node to the leftmost child node indicated that reviews were split into the leftmost child node if their flavor accuracy aspects were positive. The label on the leaf node was the predicted sentiment of reviews that belong to this node, and the number in parentheses referred to the number of reviews on the node.

Table 1 shows the normalized information gain computed with the ID3 algorithm. The aspect with higher information gain is more important. According to results, flavor accuracy and value were the two most important aspects that influence users’ sentiments toward e-liquids.

**Table 1:** Normalized information gain of each aspect.

Aspect	Normalized information gain
Flavor accuracy	0.8912
Value	0.1022
Cloud production	0.0049
Throat hit	0.0017

**Table 2:** The number of reviews for each flavor category.

Flavor	Number of reviews
Coffee	282
Cool	1609
Creamy	4056
Fruity	9653
Nutty	625
Rich	3268
Spiced	1089
Sweet	5128
Tobacco	1360

**Table 3:** Sentiment analysis of reviews for fruity and sweet flavors.

Reviews	Fruity (n=9653), n (%)	Sweet (n=5128), n (%)
Positive	6381 (66.10)	3315 (64.65)
Negative	1233 (12.77)	739 (14.41)
Neutral	2039 (21.12)	1074 (20.94)

### Statistics of Reviews for Each Flavor Category

The numbers of reviews for each flavor category are listed in Table 2. Flavors with more reviews were more popular. Table 2 shows that fruity and sweet were the two most popular categories.

Furthermore, we counted the numbers of positive, negative, and neutral reviews for these two popular flavors. As shown in Table 3, both flavors had more positive reviews than negative reviews. Sweet flavors had a higher percentage of negative reviews than fruity flavors.

### Opinion Sentiment Summarization

By mining the opinion sentiment summarizations of flavor accuracy, throat hit, value, and cloud production aspects for different flavors, decision makers and businesses have the opportunity to know why users like or dislike the aspect, thus gaining better understanding of users’ vaping experience. Multimedia Appendix 1 shows identified aspect words. Flavor-related words included “flavor,” “juice,” “vape,” “taste,” “aftertaste,” etc. Value-related words included “price,” “value,” “quality,” etc. Cloud production-related words included “vapor production,” “vapor,” “cloud production,” etc. Throat hit-related words included “throat,” “hit,” “throat hit,” etc.

Multimedia Appendix 2 shows opinion summarization of the flavor accuracy aspect for fruity and sweet flavors. Fruity flavors cover a wide range, and since different flavors have different tastes, they have the most positive and negative reviews. Users were satisfied with fruity and sweet flavors with tastes such as “great,” “sweet,” “good,” “strong,” and “nice;” “weak,” “sour,” “bad,” and “terrible” tastes made users dislike fruity flavor.

Reviews with value aspect ratings ≥4 and <3 were used to generate positive and negative opinions for value aspects, respectively. Multimedia Appendix 3 shows opinion summarization of value aspect for fruity and sweet flavors. There were more opinions about price and quality, indicating that they were two key concerns about value. Products with “great,” “good,” and “reasonable” prices can attract more user attention; “steep,” “expensive,” and “crazy” prices can make users dislike the product.

Multimedia Appendix 4 shows opinion summarization of the throat hit aspect for fruity and sweet flavors. Users liked fruity and sweet flavors with a throat hit that was “strong,” “good,” “nice,” and “perfect”; users disliked these flavors when the throat hit was “nonexistent,” “weak,” “unpleasant,” and “harsh.” Specifically, users preferred strong throat hit the most and disliked harsh throat hit the most.

**Table 4:** The number of products for each flavor category.

Flavor	Number of products
Coffee	104
Cool	459
Creamy	920
Fruity	2342
Nutty	222
Rich	1033
Spiced	439
Sweet	2049
Tobacco	490

**Table 5:** Sentiment analysis of products for fruity and sweet flavors.

Sentiment	Fruity (n=2342), n (%)	Sweet (n=2049), n (%)
Positive products (%)	1307 (55.81)	1226 (59.83)
Neutral products (%)	716 (30.57)	559 (27.28)
Negative products (%)	319 (13.62)	264 (12.88)

Multimedia Appendix 5 shows opinion summarization for the cloud production aspect for fruity and sweet flavors. Generally, users were satisfied with “great” and “good” cloud production and were not satisfied with “poor” cloud production.

### Product Statistics for Each Flavor Category

We regarded products whose average overall ratings were ≥4 as positive products, <4 and ≥3 as neutral products, and <3 as negative products. Then, we counted the number of products for each flavor category. The result is shown in Table 4.

Fruity and sweet products were the two most popular e-liquids. The sentiment distribution of products for fruity and sweet flavor is presented in Table 5. Furthermore, we extracted opinions for fruity and sweet products.

### Positive and Negative Product Opinions

The positive and negative opinions for fruity and sweet products are shown in Multimedia Appendix 6. In addition to “great flavor” and “good juice,” users also expressed their love for fruity products with “great vape.” This suggested that good vapor contributes to positive reviews of fruity products. However, negative reviews were attributed to bad tastes, which were expressed by “soapy flavor,” “odd taste,” and so on.

## Discussion

### Principal Findings

This study provides a sentiment analysis of users’ ENDS vaping experience from review sites. By analyzing influential factors and opinions, we revealed users’ e-liquid preferences. Our findings may help businesses and policy makers better understand the advantages, disadvantages, and potential health risks of e-cigarette products, thus helping them to further improve product design and provide decision-making references.

Based on results obtained by the ID3 algorithm, flavor accuracy (normalized information gain=0.8912) and value (normalized information gain=0.1022) were the two most important aspects that influence users’ sentiments toward e-liquids. For the value aspect, users were concerned with price and quality; thus, a business can attract users by providing inexpensive and high-quality products, and policy makers can develop policies to manage and monitor their price and quality.

Previous research has shown that flavor has been found to be an attractive factor to ENDS users [31,32,33]. It is broadly used in Web-based social media advertisements and offline store promotions to increase the appeal of e-cigarette products [34]. Fruity and sweet were the two most popular flavors. Users’ flavor preference closely related to positive or negative content. By using sentiment opinion summarization techniques, we could reveal more information and flavor patterns among users. Opinion summarization gave reasons users like or dislike flavors. For example, opinions such as “good/great juice” were usually adopted to express users’ positive sentiments toward e-liquids. Opinions such as “sweet/strong flavor” indicated that users liked fruity and sweet flavors because of sweet and strong tastes. The result was consistent with a previous study [35], indicating that candy-like flavors could increase the appeal to starters because they mask the heavy cigarette taste; furthermore, adding candy-like flavors could potentially be perceived as enjoyable. We found that good or great or nice juice and fruity or sweet flavor might make users dependent on or be addicted to the product. Words such as “adv (all day vape)” and “addicting/be addicted to” were used to describe these feelings. Among 8186 posts containing adjectives in the positive opinion summarization for fruity and sweet flavor, the number of posts containing “adv” and “addicting/be addicted to” were 1110 and 46, respectively. For example, some users expressed their feelings as follows: “This juice was absolutely delicious and a great adv;” “this is my adv (all day vape), i love the taste of the smooth caramel paired with the crisp green apple flavor. Very addicting!!!!;” “Sweet flavor that is nice for an ADV;” “I am addicted to this juice.”

Opinions such as “bad juice/terrible flavor/harsh throat hit” described why users disliked these flavors. E-cigarette flavorings could potentially be harmful to users. Prior research has found that the majority of users reported negative sentiments about symptoms. Negative symptom words included “dry,” “nausea,” “burn,” “hurt,” “sore,” “tingle,” “fatigue,” “sick,” “toothache,” “cough,” and “headache” [22,24]. Among 929 posts containing adjectives in the negative opinion summarization for fruity and sweet flavor, the number of posts containing negative symptoms was 38. For example, users described symptoms caused by flavors as follows: “I do get a headache from all the sweeteners if I vape too much too quickly;” “Lemon vapes give me a headache;” and “The harsh throat hit makes me cough.”

Our research shows that both attractiveness and negative symptoms of fruity and sweet products had effects on users’ health. Policy makers need to pay more attention to these products and take appropriate regulatory action to reduce health risk. For example, they may formulate a comprehensive policy to manage ingredients, dosage, and sales of such products.

**Figure 3:**
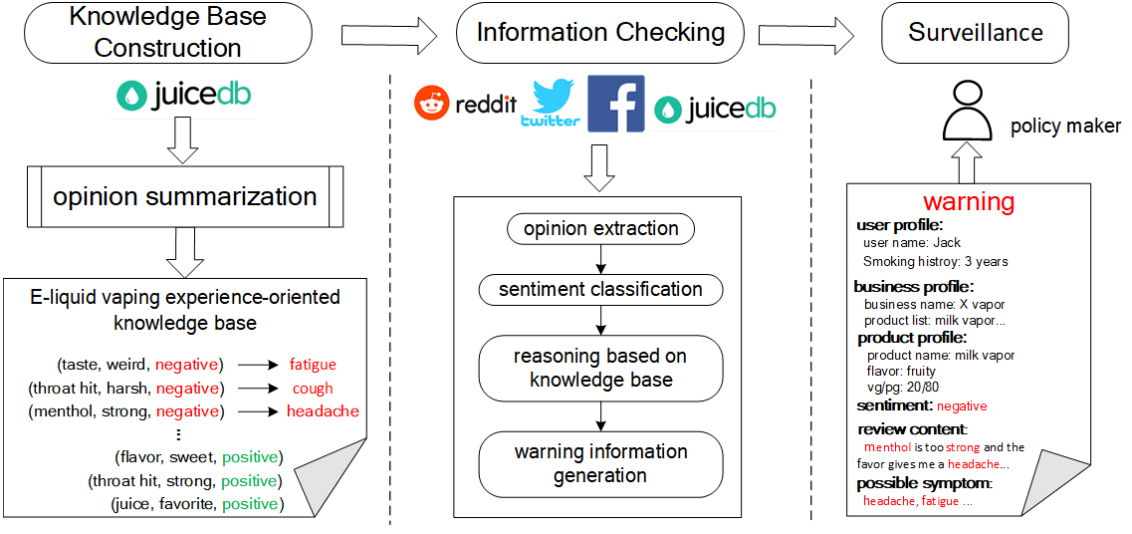
Framework showing automatic supervision of e-liquid product information.

The proposed method for analyzing vaping behavior also has the potential to be used for surveillance and detection of health-related activities on other platforms. Figure 3 shows an application scenario of the proposed framework, which can be used to monitor e-liquid product information automatically. Consider a simple example. First, we can construct an e-liquid vaping experience-oriented knowledge base, including “throat hit, harsh, negative, cough,” “menthol, strong, negative, and headache.” Furthermore, we may automatically monitor incoming information from multiple platforms including Reddit, Twitter, Facebook, and JuiceDB. When the discovery of e-liquid may be harmful to human health, the system will generate prompt warnings. For instance, incoming posts like “The menthol is strong, I feel headache” and “After vaping it all day, all week, all month, I begin to cough” will be labeled as negative, highlighted, and sent to regulatory authorities. At the same time, prevention messages could be delivered to users at risk for harm associated with e-liquid use, thus realizing automatic supervision of product information across platforms.

### Contributions

The rapid growth of ENDS, or e-cigarettes, indicates the importance of research in this field. Social media plays an indispensable role in providing new insights on e-cigarettes to help inform future research, regulations, and surveillance. Previous research has mainly utilized social media including Twitter, Facebook, YouTube, and Reddit as data sources to study e-cigarettes. Review sites such as JuiceDB provide a novel channel for users to discuss vaping methods and features; however, systematic studies on mining users’ e-liquid usage patterns from review websites are still missing. This study contributes to the field by analyzing users’ ENDS vaping experience from reviews using sentiment summarization. Specifically, we found that flavor accuracy and value were the two most important aspects that influence users’ sentiments toward e-liquids. Of reviews in JuiceDB, 67.83% (18,362/27,070) were positive, while 12.67% (3430/27,070) were negative. This indicates that users generally hold positive attitudes toward e-liquids. Among the 9 flavors, fruity and sweet were the two most popular. Great and sweet tastes, reasonable values, and strong throat hit satisfied users with “fruity” and “sweet” flavors, whereas “strange” tastes made users dislike these flavors. Meanwhile, users complained about steep or expensive prices, bad quality, and harsh throat hit of some e-liquids. There were 2342 fruity e-liquids and 2049 sweet e-liquids. There were 55.81% (1307/2342) and 59.83% (1226/2049) positive sentiments and 13.62% (319/2342) and 12.88% (264/2049) negative sentiments toward fruity e-liquids and sweet e-liquids, respectively. Great flavor and good vapor contributed to positive reviews of fruity and sweet products.

However, bad tastes such as “sour” and “bitter” resulted in negative reviews. Mined data-driven findings can help businesses and policy makers to further improve product quality and formulate effective policy.

### Limitations

We collected review data only from JuiceDB—feasible for our current research. However, several other social media platforms, such as Twitter, Facebook, and E-cigarette Forum, could be jointly used to implement cross-platform sentiment analysis.

Another limitation of this paper was incomplete demographic information. Because JuiceDB does not provide complete personal characteristics, specifically, age and gender, we could not divide our dataset into several subgroups to analyze different usage patterns among different age or gender groups.

Finally, this study used only sentiment summarization methods to mine users’ ENDS vaping experiences. Many other data mining tools could be applied to explore the dataset further. For instance, more advanced topic association methods could be adopted to discover associations between flavors and symptoms.

### Future Research

We envision three possible approaches for future study. First, the influential aspect analysis model could be extended by integrating aspect ratings and review content. In this study, we applied the ID3 algorithm to identify the relationship between aspect ratings and overall ratings; however, the review content provides more detailed semantic description information about aspect ratings. We believe that integrating these two kinds of information could produce more insights about what aspects influence users’ attitude toward e-liquid products.

Second, the aspect sentiment opinion summarization model provides basic components for analyzing aspect and product opinions. More advanced algorithms can be used to extend the model, to cluster similar opinions, and to generate more explainable opinions.

Finally, other social media platforms such as other review sites, Twitter, Reddit, etc can be considered to implement cross-platform sentiment analysis. It will be challenging and meaningful to develop a tool to monitor e-liquid product information automatically and profvide timely, valuable signals for management departments to make better decisions.

### Conclusion

This study provides an effective mechanism for analyzing users’ ENDS vaping experience based on sentiment opinion summarization techniques. Sentiment opinions for aspect and product can be found using our method, which is of great importance for monitoring e-liquid products and improving work efficiency of management departments. We hope that the characteristics we reported in this paper can be useful for other researchers and policy makers.

## Abbreviations

Adv: all day vape
API: application programming interface
ENDS: electronic nicotine delivery systems
FDA: Food and Drug Administration
ID3: iterative dichotomiser 3
PG: propylene glycol
TF: term frequency
VG: vegetable glycerin

## Appendix

Multimedia Appendix 1 Aspect words.

Multimedia Appendix 2 Opinion sentiment summarization for flavor accuracy aspect.

Multimedia Appendix 3 Opinion sentiment summarization for value aspect.

Multimedia Appendix 4 Opinion sentiment summarization for throat hit aspect.

Multimedia Appendix 5 Opinion sentiment summarization for cloud production aspect.

Multimedia Appendix 6 Opinions on products belonging to fruity and sweet categories.
