# Supplementary figures and images for: Understanding Users’ Vaping Experiences from Social Media: Initial Study Using Sentiment Opinion Summarization Techniques
Source: J Med Internet Res. 2018 Aug 15;20(8):e252. doi: 10.2196/jmir.9373 (PMC6115599; doi:10.2196/jmir.9373)

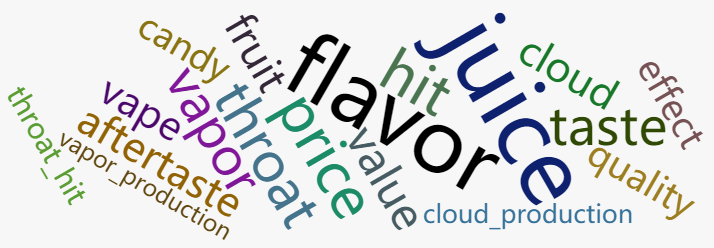

Supplement: Multimedia Appendix 1 [file jmir_v20i8e252_app1.png]

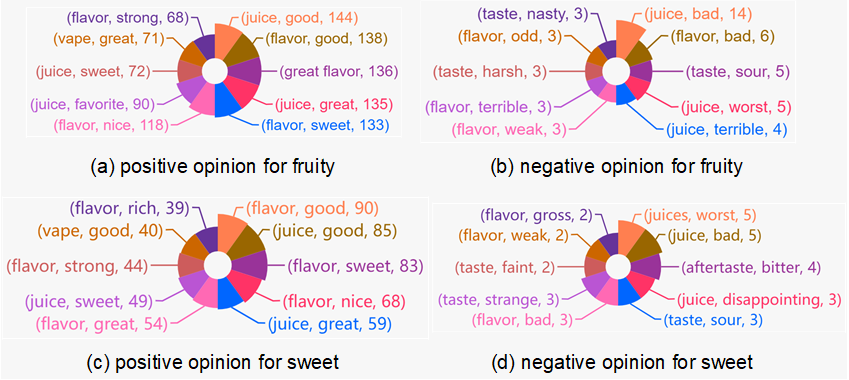

Supplement: Multimedia Appendix 2 [file jmir_v20i8e252_app2.png]

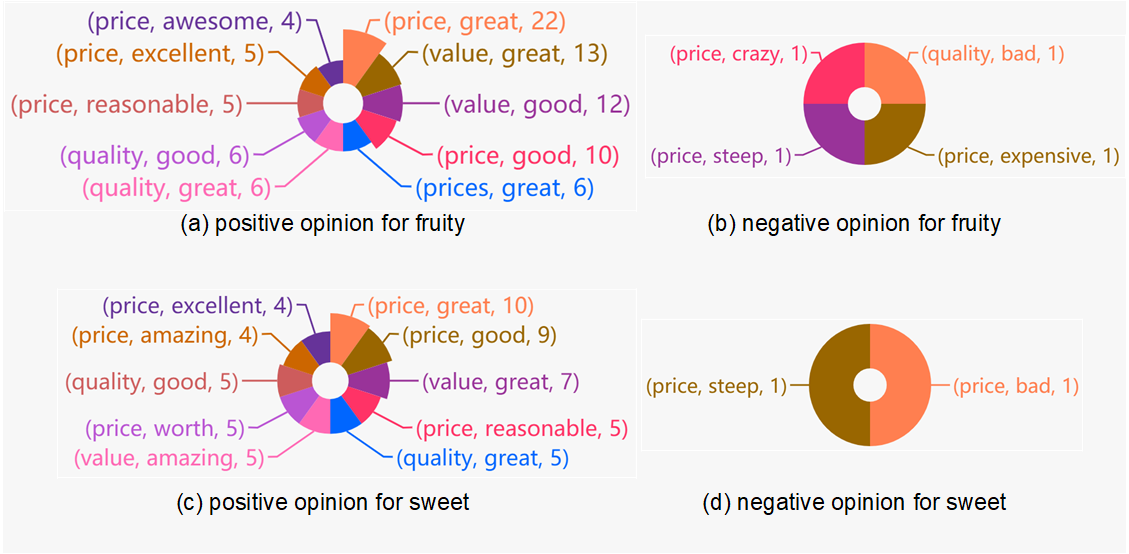

Supplement: Multimedia Appendix 3 [file jmir_v20i8e252_app3.png]

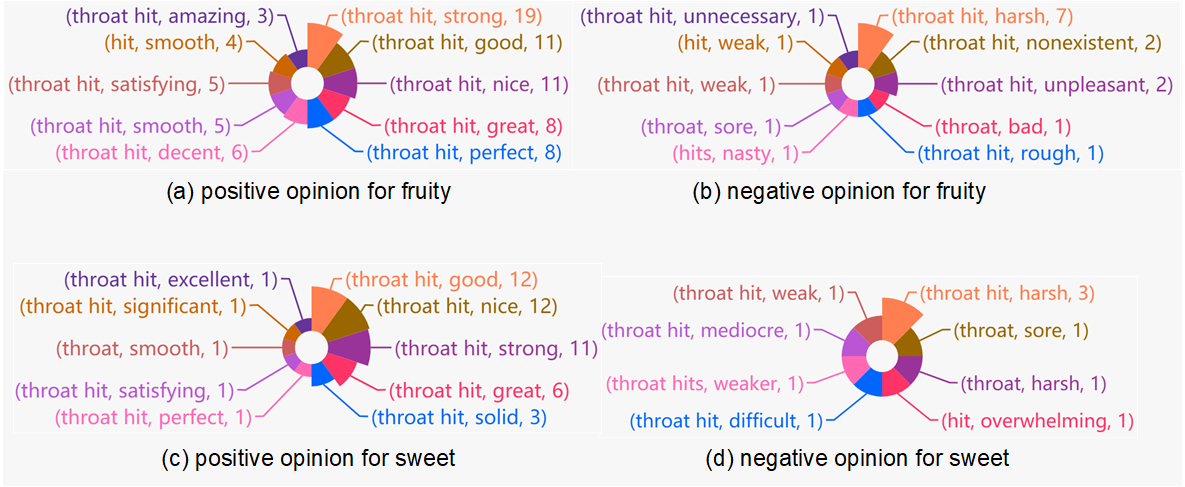

Supplement: Multimedia Appendix 4 [file jmir_v20i8e252_app4.png]

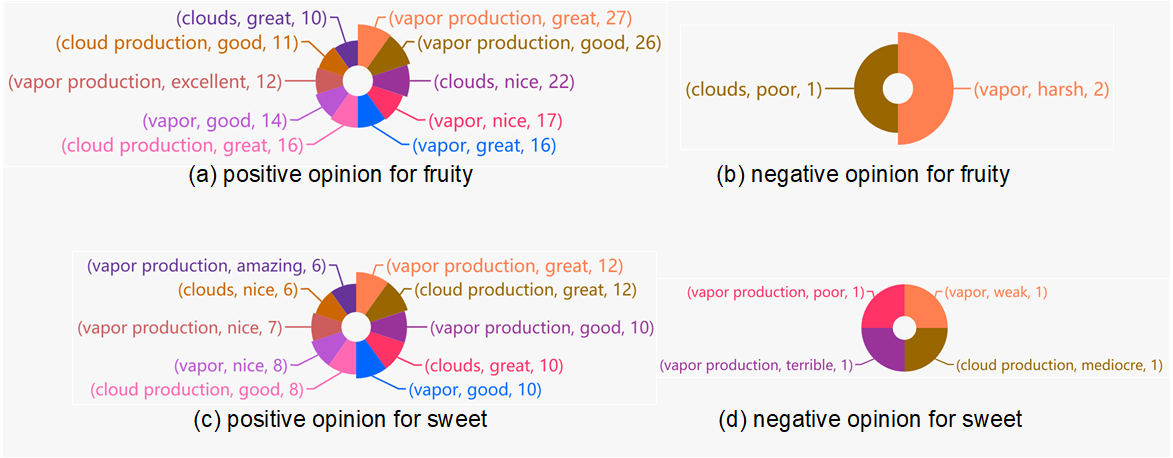

Supplement: Multimedia Appendix 5 [file jmir_v20i8e252_app5.png]

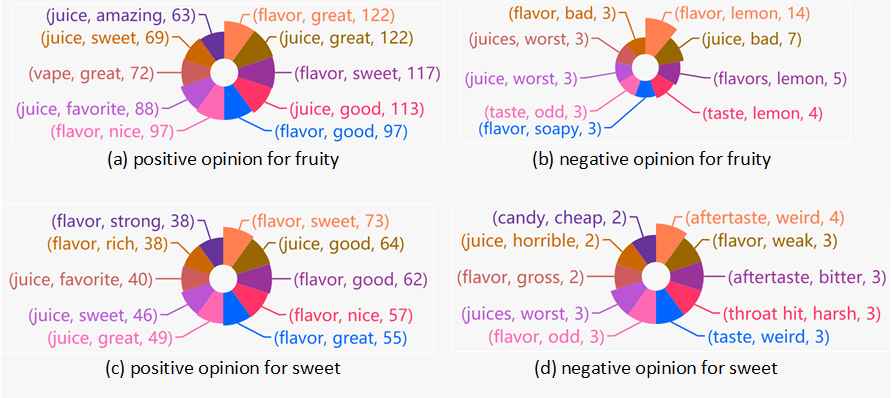

Supplement: Multimedia Appendix 6 [file jmir_v20i8e252_app6.png]
